# SGLT-2 Inhibitors and Outcomes After Transcatheter Aortic Valve Implantation: A Systematic Review and Meta-Analysis

**DOI:** 10.1016/j.jacadv.2026.102884

**Published:** 2026-06-23

**Authors:** Omer Mohammed, Kaandeeban Mohanraj, Diya Chakraborty, Seerat Kular, Sahithi Mani Chandana Paramkusam, Shelby Kutty, Negar Salehi, Aravinda Nanjundappa, Amar Krishnaswamy, Samir Kapadia, George Dangas

**Affiliations:** a Government Medical College, Kozhikode, India; b Indira Gandhi Medical College and Research Institute, Puducherry, India; c Dr Vithalrao Vikhe Patil Medical College, Ahmednagar, Maharashtra, India; d All India Institute of Medical Sciences, Bathinda, India; e Katuri Medical College and Hospital, India; f BayCare Health System, Clearwater, Florida, USA; g Cleveland Clinic Lerner College of Medicine, Cleveland, Ohio, USA; h Mount Sinai Hospital, New York, USA

**Keywords:** aortic stenosis, dapagliflozin, heart failure, SGLT-2 inhibitors, transcatheter aortic valve implantation

## Abstract

**Background:**

Despite successful transcatheter aortic valve implantation (TAVI) for severe aortic stenosis, residual heart failure risk and postprocedural complications persist.

**Objectives:**

The aim was to assess the association between sodium glucose co-transporter 2 inhibitor (SGLT-2i) use and clinical outcomes in patients undergoing TAVI.

**Methods:**

A systematic search was conducted across PubMed, Cochrane Library, Google Scholar, ScienceDirect, and ClinicalTrials.gov for eligible randomized controlled trials (RCTs) and observational studies. Adjusted HRs and risk ratios were pooled for time-to-event outcomes and dichotomous outcomes, respectively. Risk of bias was assessed using the Risk of Bias 2.0 and Risk of Bias in Non-randomized Studies of Interventions-I tools for RCTs and cohort studies, respectively.

**Results:**

Six studies (1 RCT, 5 cohorts) including 35,075 patients were included in the analysis. In pooled analyses, SGLT-2i use was associated with a significantly lower risk of major adverse cardiovascular events (pooled HR: 0.65; 95% CI: 0.44-0.95; *P* = 0.03; I^2^ = 26%), heart failure hospitalization (pooled HR: 0.58; 95% CI: 0.40-0.86; I^2^ = 59%; *P* = 0.007), all-cause mortality (pooled HR: 0.71; 95% CI: 0.55-0.91; *P* = 0.007; I^2^ = 75%), and cardiovascular death (pooled risk ratio: 0.55; 95% CI: 0.34-0.89; *P* = 0.01; I^2^ = 36%). The Grading of Recommendations Assessment, Development, and Evaluation assessment was downgraded due to heterogeneity, potential bias, and nonrandomized design of studies.

**Conclusions:**

This meta-analysis suggests that peri-operative SGLT-2i use was associated with lower cardiovascular events post-TAVI albeit with low certainty due to predominance of observational studies. These findings are hypothesis generating rather than causal inference. Future clinical trials are warranted.

Aortic stenosis (AS) is characterized by the progressive narrowing of the aortic valve, causing pressure overload–induced left ventricular hypertrophy, myocardial remodeling, and heart failure (HF).^1^ Transcatheter aortic valve implantation (TAVI) has transformed the landscape of AS management and is now the treatment of choice in symptomatic severe AS, as well as asymptomatic severe AS with left ventricular ejection fraction <50%, provided the valve and vascular anatomy are suitable.^2^ However, despite successful valve repair, postprocedural complications, such as increased incidence of arrhythmias, heart blocks, transient ischemic attacks, stroke, and acute kidney injury, put patients at risk for mortality, emphasizing the need for optimal postoperative strategies.^3^

Sodium glucose co-transporter 2 inhibitors (SGLT-2i), initially prescribed for glycemic control in diabetes mellitus, have since emerged as a cornerstone of HF management.^4^ Owing to their effect on reducing HF hospitalization and mortality, SGLT-2i are emerging as potential agents for peri-operative management of TAVI. Although mechanistic benefits of SGLT-2i in reducing upregulated SGLT-2 receptor driven inflammation, fibrosis, calcification, oxidative stress, and cardiac remodeling in AS have been demonstrated, there are limited data on improving clinical endpoints post-TAVI.^5^, ^6^, ^7^, ^8^, ^9^ The landmark DAPA-TAVI trial demonstrated a significant reduction in HF hospitalization and mortality due to these agents.^10^ Recent retrospective cohort analyses provide real-world evidence that periprocedural use of SGLT-2i reduces the risk of bioprosthetic valve failure and mortality in TAVI patients.^11^^,^^12^ The aim of this meta-analysis is to evaluate the efficacy and safety of these agents to improve long-term clinical outcomes in this high-risk population and bridge the gap in existing knowledge.

## Methods

This systematic review and meta-analysis were designed, conducted, and reported in compliance with the methodological standards of Cochrane Collaboration Handbook for Systematic Review of Interventions and the Preferred Reporting Items for Systematic Reviews and Meta-Analysis statements guidelines (Supplemental Table 1).^13^^,^^14^ The study protocol was prospectively registered in the International Prospective Register of Systematic Reviews (PROSPERO; Registration No. CRD420261277441).

### Search strategy and selection of studies

A comprehensive literature search was performed across PubMed, the Cochrane Library, Google Scholar, ScienceDirect, and clinical trials.gov utilizing the keywords “SGLT-2 inhibitor,” “empagliflozin,” “dapagliflozin,” “ertugliflozin,” “TAVI,” and “valvular heart disease” and the Boolean operators “OR” or “AND” across all databases from inception through December 27, 2025. The detailed search strategy is available on Supplemental Table 2.

Studies were included if they met the following criteria: 1) studies enrolled adult patients with AS who underwent TAVI; 2) randomized controlled trials (RCTs), nonrandomized studies, and observational studies; 3) SGLT-2i were used in the preoperative, peri-operative, or postoperative management; and 4) studies reported at least one outcome of interest. The primary outcome was the risk of major adverse cardiovascular events (MACE) post-TAVI while the secondary outcomes included risk of HF hospitalizations, all-cause death, and cardiovascular death. The inclusion and exclusion criteria are further elaborated in Supplemental Table 3.

We excluded: 1) animal studies; 2) non-SGLT-2 inhibitors as intervention; 3) prior reviews, meta-analyses, case reports, conference abstracts; 4) studies that did not report at least one outcome of interest; and 5) published in a language other than English without electronically available translation.

All the records retrieved were exported to Rayyan (Qatar Computing Research Institute)^15^ for screening. After removal of duplicates, titles and abstracts were screened by 2 independent reviewers (K.M. and D.C.) to identify potentially eligible studies. Full texts of articles deemed relevant were subsequently assessed for eligibility. Additionally, the reference lists of studies included and the relevant reviews were screened manually to identify eligible articles not retrieved by the electronic search. Any disagreements during the screening process were resolved by a third author (O.M.) through discussions and consensus.

### Data extraction

Two reviewers independently extracted data (S.M. and S.K.) from eligible studies into standardized Google Sheets form. Extracted study-level variables included country, year of publication, study design, enrollment period, sample size, and duration of follow-up. Patient-level baseline characteristics were collected separately for the SGLT-2 inhibitor and non-SGLT-2 inhibitor groups including mean age, sex distribution, prevalence of diabetes mellitus, chronic kidney disease (CKD), prior HF hospitalization, atrial fibrillation, and baseline left ventricular ejection fraction. Treatment-related data comprised timing of SGLT-2 inhibitor initiation relative to TAVI, specific agent, dosage, duration of therapy, and post-TAVI continuation status, with standard care or no SGLT-2 inhibitor as the comparator. Any disagreements during the extraction process were resolved by O.M. through discussions and consensus.

### Risk of bias assessment

Risk of bias for included studies was assessed using the Risk of Bias in Non-randomized Studies of Interventions-I and Cochrane Risk of Bias 2.0 tool.^15^^,^^16^ Plots for these were generated using the Risk-of-Bias Visualization (robvis) web application.^17^

### Data synthesis and statistical analysis

Meta-analyses were conducted using RevMan 5.4.1 and R 4.5.2.^18^^,^^19^ Adjusted HRs were pooled for time-to-event outcomes with 95% CIs using the inverse variance weighting method. Standard error was estimated using an established method (Supplemental Table 5). Cardiovascular death was treated as a dichotomous variable due to lack of time-to-event data and, hence, pooled using risk ratio (RR) using the Mantel-Haenszel method. Random-effects meta-analyses were performed due to expected clinical heterogeneity using the Der Simonian-Laird estimator with normal approximation to calculate CIs. Due to the limited number of studies, the analysis is underpowered for meta-regression or subgroup analysis. However, the source of heterogeneity was explored for the outcomes qualitatively by evaluating clinical and methodological variations across the studies and quantitatively by performing leave-one-out analysis. Sensitivity analysis was conducted excluding the pre-TAVI initiation study^20^ due to high risk of bias. Additionally, the Hartung-Knapp adjustment was applied as a sensitivity analysis to account for uncertainty from few studies. A *P* value <0.05 indicated statistical significance. Although publication bias was qualitatively assessed using funnel plots, interpretation is limited by the small number of studies (<10) (Supplemental Figure 4). The statistical tests for publication bias were not assessed due to insufficient power. Certainty of evidence was assessed using the Grading of Recommendations Assessment, Development, and Evaluation framework.^22^ The evidence was downgraded for inconsistency due to heterogeneity in study design and patient populations for all the outcomes.

## Results

### Screening and selection of studies

**Figure 1:**
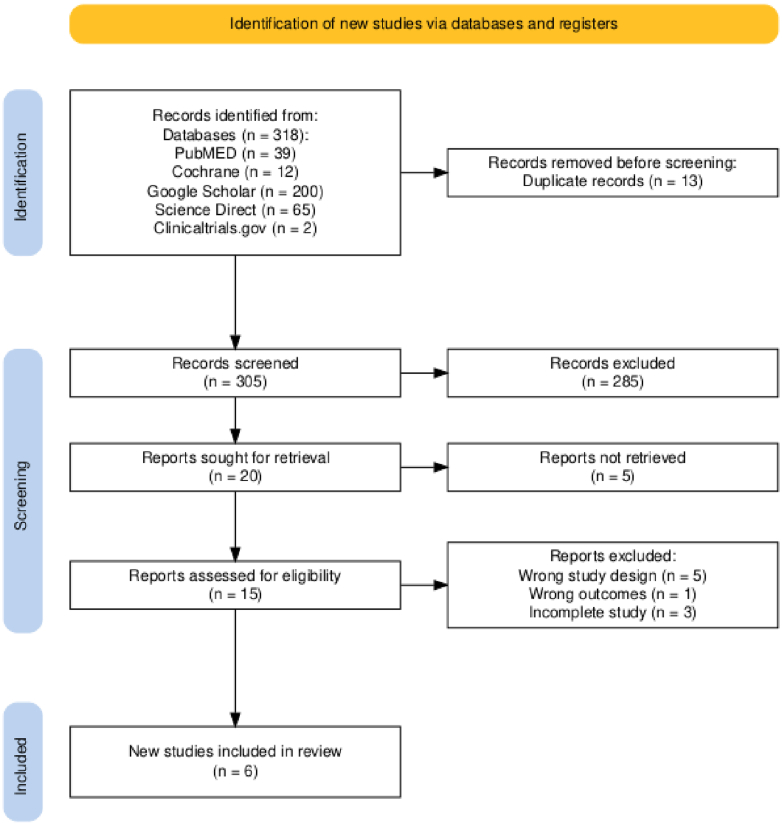
Preferred Reporting Items for Systematic Reviews and Meta-Analysis Flow Diagram for Study Selection

A total of 318 articles were retrieved from the databases searched. Following the elimination of 13 duplicates, 305 articles advanced to primary screening. After assessing the titles and abstracts, 15 articles remained for additional consideration. A subsequent full-text screening recognized 9 articles for exclusion due to either the lack of intended outcomes or improper study design or incomplete studies. Subsequently, 6 studies (1 RCT and 5 cohort studies) satisfied our inclusion criteria as shown in Figure 1.

### Baseline study characteristics

A total of 35,075 patients undergoing TAVI were analyzed (SGLT-2i = 6,207; no-SGLT-2i = 28,868). Median follow-up periods varied across studies, ranging from 6 months in the Pankaj 2024^20^ cohort to 5 years in the Omar Obeidat 2025 study.^12^ The mean age in the SGLT-2i group varied from 72.95 to 82.4 years. All studies evaluated post-TAVI use of SGLT-2i except one study which initiated SGLT-2i before TAVI.^20^ Three studies included co-existing HF patients.^10^^,^^12^^,^^20^ Four studies were predominantly male.^9^^,^^11^^,^^12^^,^^22^ One study^22^ stratified data among non-CKD and CKD subgroups and they were treated separately in the quantitative analysis as Paolisso 2025 (1) and Paolisso 2025 (2), respectively. The baseline characteristics are detailed in Tables 1 and 2.

**Table 1:** Characteristics of Studies Included in the Systematic Review and Meta-Analysis

First Author (Year)	Country	Sample Size, n (SGLT-2i/Control)	Follow-Up Duration	Type of SGLT-2i	Timing Category	Time of SGLT2i Initiation Relative to TAVI
Omar Obeidat et al (2025)^12^	United States (TrinetX)	6,044 (3,022/3,022)	Median 334.5 vs 367 d (up to 5 y)	Empagliflozin, dapagliflozin, canagliflozin, ertugliflozin	Peri-TAVI	≤1-month before or after the index TAVI procedure
Paolisso et al (2024)^9^^,^^24^	Italy	311 (74/237)	Median 24 mo (IQR 14-36)	Dapagliflozin, empagliflozin	Post-TAVI	At hospital discharge after TAVI
Raposeiras-Roubin et al (2025)^10^	Spain	1,222 (605/617)	1 y	Dapagliflozin	Post-TAVI	At hospital discharge after TAVI or ≤14 d after discharge.
Jariwala et al (2024)^23^	India	40 (20/20)	2.78/2.62 mo^a^	Empagliflozin	Pre-TAVI^b^	Prior to TAVI^b^
Paolisso et al (2025)^22^^,^^25^	Italy	514 (114/400)	Median 24 ± 15 mo	Dapagliflozin, empagliflozin	Peri-TAVI	≥4 wk prior to TAVI
Morel Olivier et al (2025)^11^	France (TriNetX, 87 countries)	26,944 (2,372/24,572)	Median 1.2 y	NR	Post-TAVI^b^	Initiated after TAVI^b^

Values are presented as number of participants or as reported in the original studies.

NR = not reported; SGLT-2i = sodium glucose co-transporter 2 inhibitors; TAVI = transcatheter aortic valve implantation.

a Values are presented as SGLT-2i group/control group.

b Timing of initiation of SGLT-2i not reported in the study.

**Table 2:** Baseline Characteristics of Study Populations

First Author (Year)	Age, y	Male (%)	Diabetes Mellitus (%)	CKD (%)	Baseline LVEF %	Heart Failure History (%)	Atrial Fibrillation (%)
Omar Obeidat et al (2025)^a^^12^	75.6 ± 8.7/75.5 ± 11.4	64.4/63.6	62.4/64.5	44.3/44.9	48.6 ± 17.6/52.1 ± 14.8	100/100	NR
Paolisso et al (2024)^9^^,^^24^	77 [73-81]/81 [77-84]	78.4/63.3	100/100	55.4/54.4	35 [26-42]/39 [32-45]	33.8/37.6	55.4/40.1
Raposeiras-Roubin et al (2025)^10^	82.4 ± 5.6/82.4 ± 5.5	50.6/50.6	43.6/44.2	NR	54.9 ± 12.3/54.8 ± 12.1	100/100	41.3/44.3
Jariwala et al (2024)^23^	72.95 ± 4.86/73.71 ± 4.85	40/40	NR	NR	56.58 ± 9.61/65.13 ± 10.68	100/100	15/20
Paolisso et al (2025) (no CKD subgroup)^22^	79 [75-82]	72.1/67.8	100	0	44 [35-55]/50 [40-60]	25.6/16.9	41.9/33.3
Paolisso et al (2025) (CKD Subgroup)^22^^,^^25^	78 [74-82]	67.6/55.3	100	100	38 [28-47]/45 [36-55]	39.4/35.5	60.6/41.9
Morel Olivier et al (2025)^a^^11^	75.9 ± 8.9/76.0 ± 8.8	60.3/60.1	70.1/72.6	NR	51.3 ± 16.0/51.9 ± 15.4	90.2/91.1	54.7/54.2

Values are presented as SGLT-2i group/control group, unless otherwise specified. Continuous variables are presented as mean ± SD or as median [IQR]; while categorical variables as (%).

CKD = chronic kidney disease; LVEF = left ventricular ejection fraction; other abbreviations as in Table 1.

a Values are presented after propensity-score matching of subjects.

### Quality of studies and risk of bias assessment

The DAPA-TAVI trial demonstrated low risk of bias across all 5 RoB 2 domains (Supplemental Figure 1).^5^ Among the 5 observational studies assessed with Risk of Bias in Non-randomized Studies of Interventions-I, one study^20^ was judged to have a serious overall risk of bias, primarily driven by residual confounding and participant selection, while the remaining studies were rated as having moderate overall risk of bias (Supplemental Figure 1). Across studies, bias was most frequently observed in the domains of confounding (D1) and selection of reported results (D7), whereas classification of interventions (D3) and outcome measurement (D6) were generally at low risk (Supplemental Figure 1).

### Major adverse cardiovascular events

**Figure 2:**
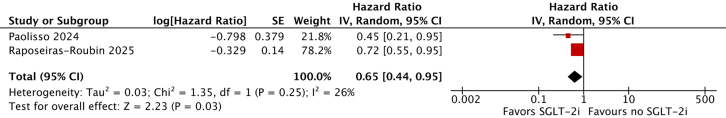
Forest Plot for Major Adverse Cardiovascular Events Forest plot for major adverse cardiovascular events comparing sodium glucose co-transporter 2 inhibitor vs control (no sodium glucose co-transporter 2 inhibitor/standard care) among patients with aortic stenosis undergoing transcatheter aortic valve implantation. IV = inverse variance; SGLT-2i = sodium glucose co-transporter 2 inhibitors.

Pooled analysis of 2 studies showed that SGLT-2i was associated with a statistically significant reduction in MACE compared to control (pooled HR: 0.65; 95% CI: 0.44-0.95; *P* = 0.03; I^2^ = 26%), as shown in Figure 2. The overall quality of evidence was rated low due to heterogeneity, differing study designs, and varying time points used to define MACE.

### HF hospitalization

**Figure 3:**
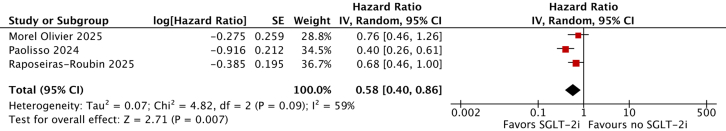
Forest Plot for Heart Failure–Related Hospitalization Forest plot for heart failure hospitalization comparing sodium glucose co-transporter 2 inhibitor vs control (no sodium glucose co-transporter 2 inhibitor/standard care) among patients with aortic stenosis undergoing transcatheter aortic valve implantation. Abbreviations as in Figure 2.

Pooled analysis of 3 studies showed SGLT-2i significantly reduced HF hospitalization compared to control (pooled HR: 0.58; 95% CI: 0.40-0.86; I^2^ = 59%; *P* = 0.007) (Figure 3). Exclusion of one study^9^ reduced I^2^ to 0 while exclusion of DAPA-TAVI trial resulted in nonsignificance (*P* = 0.06) (Supplemental Figures 2 and 3).^11^ The overall quality of evidence was rated low due to high heterogeneity, differing study designs, and potential bias in included studies (Table 3).

**Table 3:** Summary of Meta-Analysis Results

Outcomes	No. of Participants (Studies) Follow-Up	Certainty of the Evidence (GRADE)	Relative Effect HR (95% CI)
MACE	2,074 (1 RCT + 1 nonrandomized study)	⨁⨁◯◯ low	0.65 (0.44-0.95)
HF hospitalizations	6,681 (1 RCT + 2 nonrandomized studies)	⨁◯◯◯ very low	0.58 (0.40-0.86)
All-cause death	12,685 (1 RCT + 3 nonrandomized studies)	⨁◯◯◯ very low	0.71 (0.55-0.91)
Cardiovascular death	2087 (1 RCT + 4 nonrandomized studies)	⨁⨁◯◯ low	0.55 (0.34-0.89)

GRADE = Grading of Recommendations Assessment, Development, and Evaluation; HF = heart failure; MACE = major adverse cardiovascular events; RCT = randomized controlled trial; RR = risk ratio.

### All-cause death

**Figure 4:**
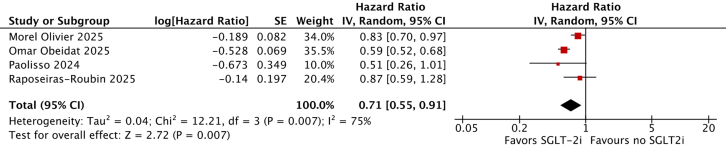
Forest Plot for All-Cause Death Forest plot for all-cause death comparing sodium glucose co-transporter 2 inhibitor vs control (no sodium glucose co-transporter 2 inhibitor/standard care) among patients with aortic stenosis undergoing transcatheter aortic valve implantation. Abbreviations as in Figure 2.

Pooled analyses of 4 studies showed a significant reduction in all-cause mortality with SGLT-2i (pooled HR: 0.71; 95% CI: 0.55-0.91; *P* = 0.007; I^2^ = 75%) (Figure 4). Exclusion of one study reduced I^2^ to 0 and resulted in the most conservative estimate (Supplemental Figures 2 and 3).^12^ The overall quality of evidence was rated low due to high heterogeneity, the nonrandomized nature of 3 studies, and moderate risk of bias in the included studies (Table 3).

### Cardiovascular death

**Figure 5:**
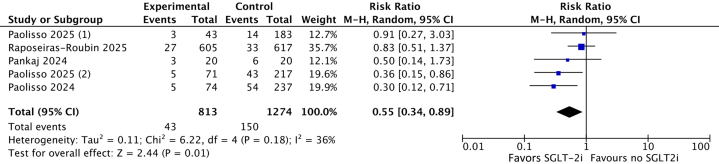
Forest Plot for Cardiovascular Death Forest plot for cardiovascular death comparing sodium glucose co-transporter 2 inhibitor vs control (no sodium glucose co-transporter 2 inhibitor/standard care) among patients with aortic stenosis undergoing transcatheter aortic valve implantation. M-H = Mantel-Haenszel; other abbreviations as in Figure 2.

Pooled analysis of 5 studies showed SGLT-2i was associated with reduced cardiovascular deaths compared to control (pooled RR: 0.55; 95% CI: 0.34-0.89; *P* = 0.01; I^2^ = 36%) (Figure 5). Statistical significance was lost when omitting either Paolisso 2024 or Paolisso 2025^11^^,^^20^ from the analysis while exclusion of DAPA-TAVI trial reduced I^2^ to 0 (Supplemental Figures 2 and 3). The overall quality of evidence was rated low due to heterogeneity, the nonrandomized nature of 3 studies, and moderate risk of bias in the included studies (Table 3).

### Sensitivity analyses

The results remained consistent after exclusion of pre-TAVI initiation study^21^ from cardiovascular death (pooled RR: 0.54; 95% CI: 0.30-0.98; *P* = 0.0009; I^2^ = 51%) (Supplemental Figure 5). Sensitivity analyses using the Hartung–Knapp adjustment showed wider CIs with attenuation of statistical significance for all outcomes, while effect estimates remained directionally consistent (Supplemental Figure 6).

### Safety

Safety outcomes were reported in the DAPA-TAVI trial.^10^ SGLT-2i were associated with a higher incidence of genital infections (1.8% vs 0.5%; *P* = 0.03) and hypotension (6.6% vs 3.6%; *P* = 0.01), whereas syncope, bacteremia, and urinary tract infections were similar between groups. No cases of ketoacidosis or necrotizing fasciitis were reported. Rates of major hypoglycemia and nontraumatic amputation were low and comparable across groups. Adverse events led to treatment discontinuation in 6.1% of patients receiving SGLT-2i in DAPA-TAVI trial.^10^

## Discussion

In contrast to prior evidence, AS is now recognized as a complex pathology of both the valve and the ventricle rather than an isolated valvular issue.^26^, ^27^, ^28^ While technological advances in TAVI devices have improved procedural success, mortality benefits related to extravalvular cardiac damage cannot be overcome just with advancing technologies, necessitating medical strategies that target the myocardium and systemic inflammation. Our pooled meta-analysis demonstrates that SGLT-2i use is associated with a significant reduction in incidence of MACE, all-cause mortality, cardiovascular deaths, and HF hospitalizations among patients following TAVI, albeit with low certainty of evidence (Central Illustration). These findings should be interpreted as hypothesis-generating given the predominance of observational data and significant residual confounding; rather than causal inference. MACE, defined as a composite of HF hospitalizations and any death, was reported at different endpoints post-TAVI across the 2 studies (DAPA-TAVI trial and Paolisso et al 2024) at 1 year and 2 years, respectively.^9^^,^^10^ HRs were pooled, which partially accounts for differing follow-up durations; however, variation in follow-up time and potential non-proportional hazards may introduce indirectness and heterogeneity, limiting the interpretation.

The leave-one-out analysis for all-cause death showed that even when the largest studies are omitted, mortality benefit was noticed, with HR ranging from 0.65 to 0.82. However, high heterogeneity (I^2^ = 75%) limits definitive interpretation. Notably, the DAPA-TAVI randomized trial did not show a standalone reduction in all-cause mortality (HR: 0.87; 95% CI: 0.59-1.28), whereas the large TriNetX registry cohort reported significant mortality benefits.^10^^,^^12^ This discrepancy is likely attributable to differing patient sample sizes and follow-up durations. While DAPA-TAVI enrolled approximately 1,222 patients with a 1-year follow-up, the Omar Obeidat et al (2025) cohort included 6,044 matched patients with longitudinal data extending up to 5 years. Furthermore, there was a marked difference in mean age, with DAPA-TAVI participants averaging 82.4 years compared to approximately 75 to 76 years in the TriNet studies (Table 2), suggesting that the benefits of SGLT-2i may be more readily detectable in a younger cohort or that a longer follow-up period than 1 year is required for the mortality curves to separate significantly.

The exclusion of DAPA-TAVI trial resulted in a nonsignificant reduction of HF hospitalizations in our meta-analysis (Supplemental Figure 2). This is due to residual confounding in the study by Morel Olivier et al^11^ (2025) which likely has a higher proportion of patients with heart failure with preserved ejection fraction or low-flow low gradient AS, which are frequent indications to start SGLT-2i therapy. The findings are confirmed by exclusion of the latter study, which resulted in the greatest reduction in HF hospitalization (pooled HR: 0.53; 95% CI: 0.31-0.88) (Supplemental Figure 2). While these studies had a high proportion of patients with previous HF hospitalization, only a third (36.7%) of patients in Paolisso et al (2024) had prior HF hospitalization, which explains the reduction of I^2^ to 0 by exclusion of the latter study (Supplemental Figure 3).

Cardiovascular deaths were reduced in the SGLT-2i group despite the dominant study (DAPA-TAVI) having nonsignificant reduction. Sensitivity analysis revealed exclusion of either (or both) of 2 studies resulted in loss of significance (Supplemental Figure 2).^9^^,^^22^ These studies had higher prevalence of prior coronary artery disease or myocardial infarction compared to the others (Table 2). Although long-term data are limited, recent meta-analyses have shown that SGLT-2i reduces cardiovascular events post-myocardial infarction^29^ and hence, these studies derived likely benefit from their use.

Sensitivity analyses using the Hartung–Knapp adjustment highlighted the influence of study number on statistical significance. While this conservative method is preferred, it may lack power with few studies. Nonetheless, the consistent effect direction and substantial patient population suggest a plausible benefit of SGLT-2i in TAVI patients.

### Biological plausibility

Although normal myocardium shows minimal SGLT-2 expression, pressure overload and myocardial hypertrophy, as seen in AS, can upregulate the expression of SGLT-2, peroxisome proliferator-activated receptor-γ, and simultaneous downregulation peroxisome proliferator-activated receptor-α expression.^5^ This shifts the cellular energetics favoring glucose metabolism over fatty acid oxidation, leading to lipid accumulation and lipotoxicity.^6^ Accumulated lipids activate the innate immunity via Toll-like receptors and nuclear factor-kappa B, leading to an increase in interleukin-6, driving inflammation and oxidative stress.^6^^,^^7^

Increased intracellular sodium mediated by SGLT-2 disrupts mitochondrial calcium handling via the Na^+^/Ca^2+^ exchanger, causing calcium overload and reactive oxygen species generation.^6^^,^^8^ Reactive oxygen species upregulate bone morphogenetic proteins and transforming growth factor-beta (TGF-β), promoting valvular calcification and osteogenesis.^8^^,^^30^ Chronic left ventricle mechanical strain further activates latent TGF-β through integrin-mediated conformational changes, sustaining TGF-β signaling without de novo synthesis.^5^^,^^7^^,^^8^ This, in addition to inflammation and oxidative damage, drives fibrosis, calcification, and cardiac remodeling.^5^^,^^7^^,^^8^ In the context of TAVI where the native, diseased valve remains in situ, these pathways may plausibly influence the surrounding inflammatory and fibrotic milieu. However, the potential benefits of SGLT-2i on native valve-bioprosthetic valve interactions, valvular degeneration, or long-term durability remain speculative and have not been directly demonstrated. A recent observational study reported a lower annual rate of valve failure (0.75% vs 1.34%) suggesting that SGLT-2 inhibitors may have a direct disease-modifying effect on the bioprosthesis by attenuating the pro-inflammatory environment that promotes valve degeneration.^11^ However, further prospective validation is required to validate these effects.

A further mechanistic dimension pertains to coronary microvascular dysfunction and its interplay with SGLT-2i. TAVI improves coronary microvascular perfusion over time by relieving pressure overload; however, coronary microvascular dysfunction independently perpetuates myocardial damage in AS and may persist despite hemodynamic relief.^24^^,^^31^ SGLT-2i have been shown to improve microvascular perfusion and reduce microvascular inflammation, which may represent an additional complementary mechanism through which these agents attenuate ongoing cardiac damage beyond the procedural benefits of TAVI itself.^32^

### Strengths and limitations

To our knowledge, this is the first and largest meta-analysis to date that assessed the impact of SGLT-2 inhibitors in preoperative and postoperative management of TAVI. This study pooled evidence across 35,075 patients from the landmark DAPA-TAVI trial alongside large-scale, real-world data from multicenter registries to provide real-world evidence. However, several limitations must be considered when interpreting these findings. First, the majority of the included evidence (5 out of 6 studies) stems from retrospective observational designs, which are inherently susceptible to residual confounding, selection bias, and treatment-indication bias despite the use of rigorous propensity score matching. SGLT-2i are preferentially prescribed to patients with specific cardiometabolic profiles, which may overstate observed treatment associations. Second, a significant limitation of these studies involves the reliance on International Classification of Diseases, 10th Revision diagnostic coding for defining clinical endpoints. This approach lacks the granularity of core-laboratory adjudicated data and may fail to capture subclinical bioprosthetic valve dysfunction or hemodynamic changes that do not result in a formal diagnosis. Third, medication exposure was primarily determined by prescription records, which do not necessarily reflect long-term patient adherence or treatment duration. Fourth, key procedural factors, such as valve type (balloon-expandable vs self-expanding), commissural alignment, residual aortic regurgitation, pacemaker implantation rates, and procedural complications were largely unavailable in the registry data sets and not accounted for in pooled analyses, limiting insights into the mechanical factors influencing valve durability despite their strong independent influence on post-TAVI outcomes. Fifth, the degree of clinical and methodological heterogeneity is substantial. Studies varied widely in timing of SGLT-2i initiation, patient populations (differing HF burden and CKD prevalence), and follow-up durations (6 months to 5 years), reflected in high I^2^ values. Sixth, 4 studies had high proportion of elderly males, the age group most likely to receive TAVI, and hence the findings cannot be generalized to young adults or females planned for TAVI. Seventh, a subgroup analysis for low-flow low-gradient AS could not be performed due to insufficient granularity of the available data. Eighth, a formal pooled analysis of safety outcomes including acute kidney injury, hypotension, volume depletion, euglycemic ketoacidosis, and genitourinary infections was not feasible due to inconsistent reporting across studies; this represents a critical evidence gap given the elderly, frail, and volume-sensitive nature of the TAVI population. These factors may influence tolerability and account to drug discontinuation leading to nonefficacy. SGLT-2i–induced osmotic diuresis and mild hypokalemia may prompt initiation of mineralocorticoid receptor antagonists such as spironolactone, potentially confounding the observed benefits. Although sensitivity analysis was conducted to explore the source of heterogeneity, our analysis is underpowered for subgroup analysis or meta-regression. Due to few studies, interpretation of publication bias is limited. Finally, the study populations were largely drawn from high-income countries, which may limit the generalizability of these results to underrepresented geographic regions.

### Future directions

Future research should prioritize large-scale, prospective randomized trials with extended follow-up periods to definitively establish the mortality benefits and long-term valve-protective effects of SGLT-2 inhibitors. Given that current evidence is heavily weighted toward diabetic populations, it is crucial to investigate whether these cardiovascular and renal benefits extend to nondiabetic TAVI recipients. Stratification by age, frailty status, and flow-gradient pattern is recommended. Additionally, future studies should incorporate multimodal imaging, such as 18F-sodium fluoride positron emission tomography, to validate the hypothesized disease-modifying role of SGLT-2i in mitigating native-valve and bioprosthetic interactions. Further investigation is also required to determine the optimal timing of SGLT-2i initiation (periprocedural vs long-term maintenance) and to refine patient selection criteria for those most likely to benefit from this adjunctive therapy. The evolving landscape of TAVI, particularly its expansion to younger and lower-risk populations, further elevates the importance of adjunctive medical therapies aimed at preserving long-term outcomes.^25^ In this context, structured heart valve clinics represent an important framework for longitudinal medication management, complication surveillance, and optimization of pharmacological strategies including SGLT-2i in post-TAVI patients.^33^

## Conclusions

The use of SGLT-2 inhibitors in the preoperative and postoperative setting of TAVI was associated with a reduction in the risk of all-cause mortality, HF hospitalizations, cardiovascular deaths, and MACE, albeit with a low certainty of evidence. Given the predominance of observational data, causality cannot be established, and these findings should be considered exploratory in nature. Future RCTs with adequate power are needed to validate these associations.

Perspectives **COMPETENCY IN PRACTICE-BASED LEARNING:** This systematic review and meta-analysis demonstrated that SGLT-2 inhibitors were associated with lower MACE following TAVI. Given the predominance of observational data and low certainty, findings are hypothesis-generating and need to be validated by future RCTs.

**TRANSLATIONAL OUTLOOK:** This study explores the potential role of SGLT-2 inhibitors in peri-operative TAVI management by blocking the pro-inflammatory effects of SGLT-2 up-regulation in myocardium and native valve in AS patients.

**Central Illustration:**
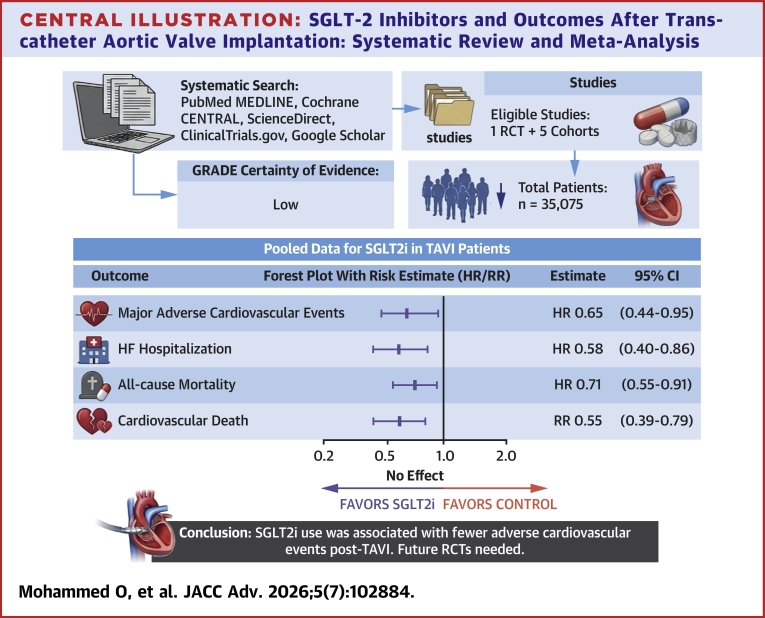
SGLT-2 Inhibitors and Outcomes After Transcatheter Aortic Valve Implantation: Systematic Review and Meta-Analysis This systematic review and meta-analysis evaluated the association between sodium glucose co-transporter 2 inhibitor use and clinical outcomes in patients undergoing transcatheter aortic valve implantation across 35,075 patients in 6 studies. Sodium glucose co-transporter 2 inhibitor use was associated with lower cardiovascular events and mortality post-transcatheter aortic valve implantation albeit with low certainty due to predominance of observational studies. HF = heart failure; RCT = randomized controlled trials; RR = risk ratio; TAVI = transcatheter aortic valve implantation; other abbreviation as in Figure 2.

## Funding support and author disclosures

The authors have reported that they have no relationships relevant to the contents of this paper to disclose.
