# Supplementary material for: SGLT-2 Inhibitors and Outcomes After Transcatheter Aortic Valve Implantation: A Systematic Review and Meta-Analysis
Source: JACC Adv. 2026 Jun 23;5(7):102884. doi: 10.1016/j.jacadv.2026.102884 (PMC13320029; doi:10.1016/j.jacadv.2026.102884)

**Supplemental appendix**

Supplemental Table S1: PRISMA Checklist

Supplemental Table S2: Advanced Search Strategy

Supplemental Table S3: Inclusion and Exclusion Criteria

Supplemental Table S4: Outcome Definition across Studies

Supplemental Table S5: Estimation of Standard Error from Hazard Ratio with 95% Confidence Intervals

Supplemental Material: Protocol

Supplemental Figure S1: Risk of Bias Assessment

Supplemental Figure S2: Leave One Out Analysis Plots

Supplemental Figure S3: Sensitivity Analyses Plots for Heterogeneity Assessment

Supplemental Figure S4: Funnel Plots for Publication Bias

Supplemental Figure S5: Sensitivity Analyses Plots After Excluding Pre-TAVI SGLT2i initiation study

Supplemental Figure S6: Sensitivity Analyses Plots After Hartung-Knapp Adjustment

**Supplemental Table S1: Prisma Checklist**

| **Section and Topic** | **Item #** | **Checklist item** | **Location where item is reported** |
| --- | --- | --- | --- |
| **TITLE** | | |  |
| Title | 1 | Identify the report as a systematic review. | 1 |
| **ABSTRACT** | | |  |
| Abstract | 2 | Provide an explicit statement of the main objectives, inclusion and exclusion criteria, information sources, risk of bias, methods to synthesise results, participants, limitations of evidence, interpretation of results, primary source of funding, and registration number. | 2,3 |
| **INTRODUCTION** | | |  |
| Rationale | 3 | Describe the rationale for the review in the context of existing knowledge. | 5 |
| Objectives | 4 | Provide an explicit statement of the objective(s) or question(s) the review addresses. | 5 |
| **METHODS** | | |  |
| Eligibility criteria | 5 | Specify the inclusion and exclusion criteria for the review and how studies were grouped for the syntheses. | 6, Table S3 |
| Information sources | 6 | Specify all databases, registers, websites, organisations, reference lists and other sources searched or consulted to identify studies. Specify the date when each source was last searched or consulted. | 6 |
| Search strategy | 7 | Present the full search strategies for all databases, registers and websites, including any filters and limits used. | Table S2 |
| Selection process | 8 | Specify the methods used to decide whether a study met the inclusion criteria of the review, including how many reviewers screened each record and each report retrieved, whether they worked independently, and if applicable, details of automation tools used in the process. | 6,7 |
| Data collection process | 9 | Specify the methods used to collect data from reports, including how many reviewers collected data from each report, whether they worked independently, any processes for obtaining or confirming data from study investigators, and if applicable, details of automation tools used in the process. | 7 |
| Data items | 10a | List and define all outcomes for which data were sought. Specify whether all results that were compatible with each outcome domain in each study were sought (e.g. for all measures, time points, analyses), and if not, the methods used to decide which results to collect. | 7 |
|  | 10b | List and define all other variables for which data were sought (e.g. participant and intervention characteristics, funding sources). Describe any assumptions made about any missing or unclear information. | 7 |
| Study risk of bias assessment | 11 | Specify the methods used to assess risk of bias in the included studies, including details of the tool(s) used, how many reviewers assessed each study and whether they worked independently, and if applicable, details of automation tools used in the process. | 7,8 |
| Effect measures | 12 | Specify for each outcome the effect measure(s) (e.g. risk ratio, mean difference) used in the synthesis or presentation of results. | 8 |
| Synthesis methods | 13a | Describe the processes used to decide which studies were eligible for each synthesis (e.g. tabulating the study intervention characteristics and comparing against the planned groups for each synthesis (item #5)). | 8 |
|  | 13b | Describe any methods required to prepare the data for presentation or synthesis, such as handling of missing summary statistics, or data conversions. | 8 |
|  | 13c | Describe any methods used to tabulate or visually display results of individual studies and syntheses. | 8 |
|  | 13d | Describe any methods used to synthesize results and provide a rationale for the choice(s). If meta-analysis was performed, describe the model(s), method(s) to identify the presence and extent of statistical heterogeneity, and software package(s) used. | 8 |
|  | 13e | Describe any methods used to explore possible causes of heterogeneity among study results (e.g. subgroup analysis, meta-regression). | 8 |
|  | 13f | Describe any sensitivity analyses conducted to assess robustness of the synthesized results. | 8 |
| Reporting bias assessment | 14 | Describe any methods used to assess risk of bias due to missing results in a synthesis (arising from reporting biases). | 8 |
| Certainty assessment | 15 | Describe any methods used to assess certainty (or confidence) in the body of evidence for an outcome. | 8 |
| **RESULTS** | | |  |
| Study selection | 16a | Describe the results of the search and selection process, from the number of records identified in the search to the number of studies included in the review, ideally using a flow diagram. | 9 |
|  | 16b | Cite studies that might appear to meet the inclusion criteria, but which were excluded, and explain why they were excluded. | - |
| Study characteristics | 17 | Cite each included study and present its characteristics. | 9, Tables 1, 2 |
| Risk of bias in studies | 18 | Present assessments of risk of bias for each included study. | 9, Figure S1 |
| Results of individual studies | 19 | For all outcomes, present, for each study: (a) summary statistics for each group (where appropriate) and (b) an effect estimate and its precision (e.g. confidence/credible interval), ideally using structured tables or plots. | - |
| Results of syntheses | 20a | For each synthesis, briefly summarise the characteristics and risk of bias among contributing studies. | 10-11 |
|  | 20b | Present results of all statistical syntheses conducted. If meta-analysis was done, present for each the summary estimate and its precision (e.g. confidence/credible interval) and measures of statistical heterogeneity. If comparing groups, describe the direction of the effect. | 10-11 |
|  | 20c | Present results of all investigations of possible causes of heterogeneity among study results. | 10-11, Figures S2,S3 |
|  | 20d | Present results of all sensitivity analyses conducted to assess the robustness of the synthesized results. | Figures S5, S6 |
| Reporting biases | 21 | Present assessments of risk of bias due to missing results (arising from reporting biases) for each synthesis assessed. | Figure S4 |
| Certainty of evidence | 22 | Present assessments of certainty (or confidence) in the body of evidence for each outcome assessed. | Table 3 |
| **DISCUSSION** | | |  |
| Discussion | 23a | Provide a general interpretation of the results in the context of other evidence. | 12 |
|  | 23b | Discuss any limitations of the evidence included in the review. | 15-16 |
|  | 23c | Discuss any limitations of the review processes used. | 15-16 |
|  | 23d | Discuss implications of the results for practice, policy, and future research. | 12-16 |
| **OTHER INFORMATION** | | |  |
| Registration and protocol | 24a | Provide registration information for the review, including register name and registration number, or state that the review was not registered. | 6 |
|  | 24b | Indicate where the review protocol can be accessed, or state that a protocol was not prepared. | 6 |
|  | 24c | Describe and explain any amendments to information provided at registration or in the protocol. | S9, S10 |
| Support | 25 | Describe sources of financial or non-financial support for the review, and the role of the funders or sponsors in the review. | 18 |
| Competing interests | 26 | Declare any competing interests of review authors. | 18 |
| Availability of data, code, and other materials | 27 | Report which of the following are publicly available and where they can be found: template data collection forms; data extracted from included studies; data used for all analyses; analytic code; any other materials used in the review. | 18 |

Supplemental Table S1 provides the PRISMA 2020 Checklist, detailing the adherence of the study to each reporting item required for systematic reviews and meta-analyses

**Supplemental Table S2: Advanced Search Strategy**

Databases Included:

1. PubMed including MEDLINE
2. Cochrane Central Library
3. Google Scholar
4. ScienceDirect
5. Google Scholar
6. Clinical Trials.gov

| Database | Search Strategy | Results |
| --- | --- | --- |
| PubMed(Including MEDLINE) | ( "Sodium-Glucose Transporter 2 Inhibitors"[Mesh] OR empagliflozin[Title/Abstract] OR dapagliflozin[Title/Abstract] OR canagliflozin[Title/Abstract] OR ertugliflozin[Title/Abstract] OR "SGLT2 inhibitor*"[Title/Abstract] ) AND ( "Transcatheter Aortic Valve Replacement"[Mesh] OR TAVR[Title/Abstract] OR TAVI[Title/Abstract] OR "transcatheter aortic valve"[Title/Abstract] OR "aortic stenosis"[Mesh] OR "aortic stenosis"[Title/Abstract] ) | 39 |
| Cochrane | ("SGLT 2 inhibitors" OR "dapagliflozin" OR "empagliflozin" OR "canagliflozin") AND ("transcatheter aortic valve replacement" OR "TAVR" OR "TAVI" OR "aortic stenosis" OR "aortic sclerosis") | 12  Title and Abstract keywords |
| Google Scholar | (“empagliflozin” OR “dapagliflozin” OR “canagliflozin” OR “sglt2 inhibitors”) AND (“tavi” OR “tavr” OR “transcatheter aortic valve” OR “aortic stenosis”) | 200 |
| Science Direct | (“empagliflozin” OR “dapagliflozin” OR “canagliflozin” OR ”sglt2 inhibitors”) AND (“tavi” OR “tavr” OR “transcatheter aortic valve”) | 65  FILTERS APPLIED: Research Articles  English |
| Clinical Trials.gov | (“dapagliflozin”) AND (“TAVI” OR “TAVR” OR “transcatheter aortic valve” OR (“aortic stenosis”)) | 2  FILTERS APPLIED: Completed Trials |

Outlines the comprehensive search terms, Boolean operators, and database-specific syntax used to identify relevant studies across multiple electronic databases.

**Supplemental Table S3: Inclusion and Exclusion Criteria**

| **INCLUSION CRITERIA** | **EXCLUSION CRITERIA** |
| --- | --- |
| **Population:** Adults (≥18 years) with aortic stenosis who underwent TAVR | **Population:** Non-human studies |
| **Study Design:** Randomized controlled trials, non-randomized studies, observational studies | **Study Design:** Systematic reviews, meta-analyses, narrative reviews, case reports/series, editorials |
| **Intervention:** Any SGLT-2 inhibitor (empagliflozin, dapagliflozin, canagliflozin, ertugliflozin etc.) | **Intervention:** Studies evaluating drugs other than SGLT-2 inhibitors as the primary intervention |
| **Comparator:** Placebo or standard care | **Comparator:** Studies without a comparator arm |
| **Outcomes:**  Major adverse cardiovascular events, HF hospitalization, all-cause mortality, CV death | Studies without sufficient data on outcomes. |
| **Period:** Studies published from inception |  |

**Supplemental Table S4: Outcome Definition across Studies**

| **Study** | **MACE Definition** | **HF Hospitalization Definition** | **CV Death Definition** | **First vs Recurrent Events** | **Adjudication Method** | **Follow-up Duration** | **Outcome Type** |
| --- | --- | --- | --- | --- | --- | --- | --- |
| Omar Obeidat 2025 | Not defined | EHR-based ICD coding for HF hospitalization | Not defined | First-event analysis | EHR-based on ICD codes | 5 yrs | Time-to-first event |
| Paolisso 2024 | Composite of cardiovascular mortality and non-fatal heart failure hospitalization | Diagnosed as per current ESC 2023 guidelines | Deaths resulting from an AMI, sudden cardiac death, heart failure, stroke, and other cardiovascular causes | First-event analysis | Registry-based. | 2 years | Time-to-first event |
| Paolisso 2025 | Composite of cardiovascular mortality and non-fatal heart failure hospitalization | Diagnosed as per current ESC 2023 guidelines | Deaths resulting from an AMI, sudden cardiac death, heart failure, stroke, and other cardiovascular causes | First-event analysis | Registry-based | 2 years | Time-to-first event |
| Raposeiras-Roubín 2025 (DapaTAVI) | Composite of death from any cause or worsening of heart failure, defined as hospitalization or an urgent visit, at 1 year of follow-up. | Worsening HF defined as hospitalization or urgent visit requiring IV diuretics | Death from cardiovascular causes (adjudicated) | First-event analyses. Recurrent events analyses used for total HF hospitalizations or CV death | Independently adjudicated clinical events committee | 1 year | Time-to-first event |
| Pankaj 2024 | Not defined | HF hospitalization (not explicitly standardized; EMR-based) | Not defined | Event-based (not clearly time-to-event) | Patient health records-based | 6 months | Cumulative incidence |

Abbreviations: **MACE** – Major Adverse Cardiovascular Events, **HF –** Heart Failure, **CV** – Cardiovascular, **EHR** – Electronic Health Records, I**CD** – International Classification of Diseases, **ESC** – European Society of Cardiology, **AMI** – Acute Myocardial Infarction, **IV** – Intravenous

**Supplemental Material: Protocol**

**REVIEW TITLE AND BASIC DETAILS**

**Review title**

Efficacy of sodium–glucose cotransporter-2 inhibitors in patients undergoing transcatheter aortic valve replacement: a systematic review and meta-analysis

**Condition or domain being studied**

Sodium-glucose Co-transporter 2 (SGLT2) Inhibitors

**Rationale for the review**

Transcatheter aortic valve replacement (TAVR) is the standard of care for patients with severe aortic stenosis across surgical risk categories. Despite procedural success, post-TAVR patients remain at high risk for heart failure hospitalization, renal dysfunction, and mortality. Sodium–glucose cotransporter-2 (SGLT2) inhibitors have demonstrated robust benefits in reducing heart failure events and improving renal outcomes across a broad spectrum of cardiovascular disease, independent of diabetes status. Emerging observational data and post-hoc analyses suggest potential benefits of SGLT2 inhibitors in patients undergoing TAVR; however, the efficacy and safety of SGLT2 inhibitor therapy initiated before or continued after TAVR have not been systematically evaluated. A comprehensive synthesis of available evidence is warranted to inform clinical decision-making.

**Review objectives**

To evaluate the efficacy and safety of SGLT2 inhibitor therapy in patients following transcatheter aortic valve replacement compared with standard care or non-SGLT2 inhibitor therapy.

**Keywords**

TAVR; SGLT2 inhibitors; Transcatheter aortic valve replacement; TAVR

**ELIGIBILITY CRITERIA**

**Population**

Included Adults (≥18 years) who have undergone transcatheter aortic valve replacement for severe aortic stenosis.

**Intervention(s) or exposure(s)**

Sodium-glucose Co-transporter 2 (SGLT2) Inhibitors

**Comparator(s) or control(s)**

Placebo or usual care

**Study design**

Both randomized and non-randomized study types will be included.

**AVAILABILITY OF FULL PROTOCOL**

A full protocol has been written and uploaded to PROSPERO (CRD420261277441).

**SEARCHING AND SCREENING**

**Search for unpublished studies**

Only published studies will be sought.

**Main bibliographic databases that will be searched**

The main databases to be searched are MEDLINE and PubMed.

Other important or specialist databases that will be searched Science Direct, Cochrane CENTRAL, Clinicaltrials.gov, Google scholar

**Search language restrictions**

The review will only include studies published in English.

**Search date restrictions**

Databases will be searched for articles published before 31 December 2025, there are no restrictions on search start date.

**Other methods of identifying studies**

Other studies will be identified by: searching trial or study registers.

**Selection process**

Studies will be screened independently by at least two people (or person/machine combination) with a process to resolve differences.

**Other relevant information about searching and screening**

None

**DATA COLLECTION PROCESS**

**Data extraction from published articles and reports**

Data will be extracted independently by at least two people (or person/machine combination) with a process to resolve differences. Authors will be asked to provide any required data not available in published reports.

**Study risk of bias or quality assessment**

Risk of bias will be assessed using: Cochrane RoB-2 and ROBINS-I

Data will be assessed independently by at least two people with a process to resolve differences.

**Reporting bias assessment**

Risk of bias due to missing results will be assessed

**Certainty assessment**

The GRADE framework will be used to assess the certainty of evidence for primary and secondary outcomes.

**OUTCOMES TO BE ANALYSED**

**Main outcomes**

MACE as reported in study

**Additional outcomes**

- HF hospitalization, all-cause death, CV death

**PLANNED DATA SYNTHESIS**

**Strategy for data synthesis**

Dichotomous outcomes will be pooled using risk ratios (RR) for fixed-time events or hazard ratios (HR) with 95% confidence intervals for time-to-event data. Continuous outcomes will be pooled using mean difference (MD) or standardized mean difference (SMD). A random-effects model will be used due to expected clinical heterogeneity. Heterogeneity will be assessed using the I² statistic.

**Supplemental Table S5: Estimation of Standard Error from Hazard Ratio with 95% Confidence Intervals**

| For Hazard Ratio with 95% CI, standard error is calculated as:  **Standard Error (SE) = ln (Upper CI limit) – ln (Lower CI limit)/3.92** |
| --- |

**Supplemental Figure S1: Risk of Bias Assessment**


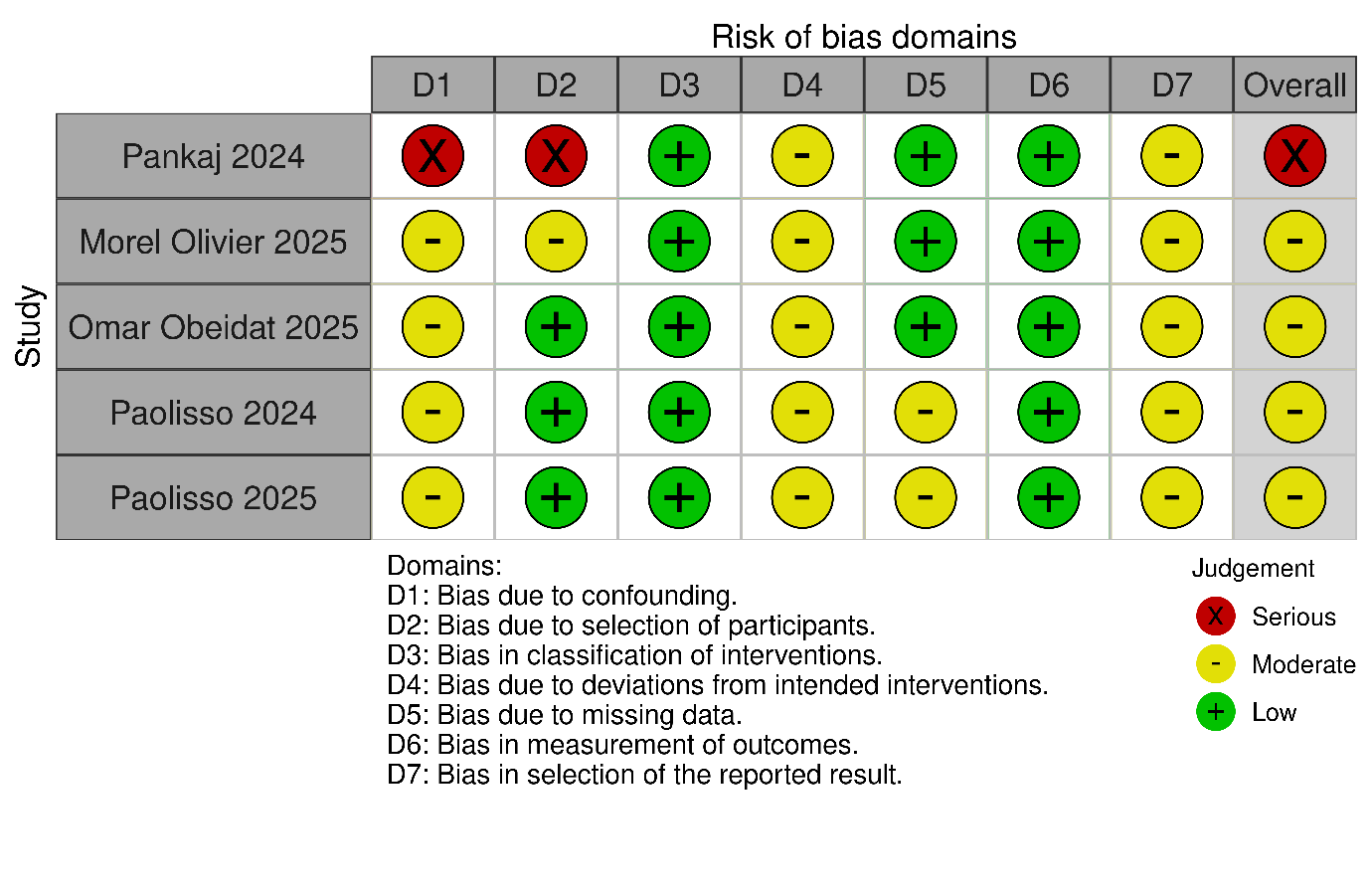


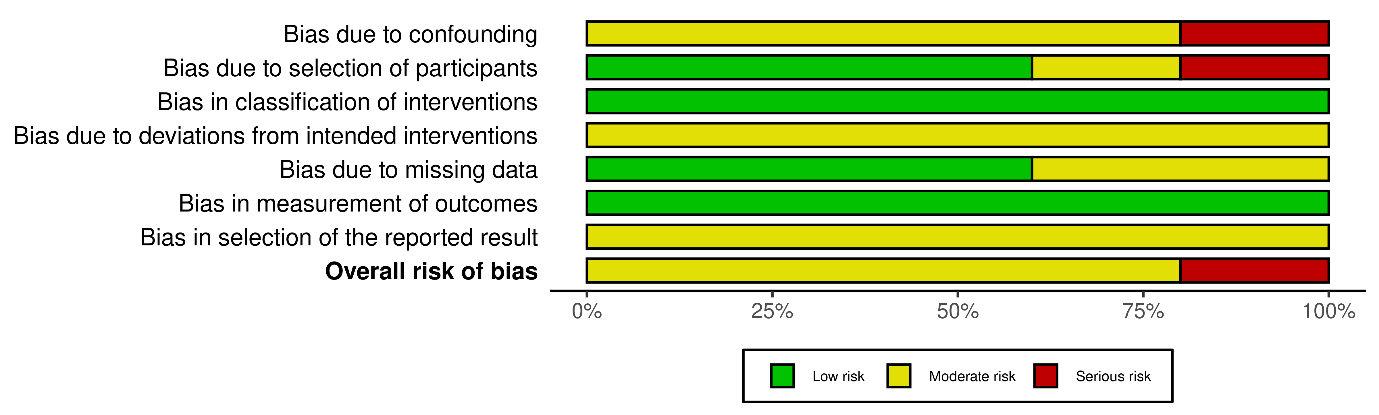


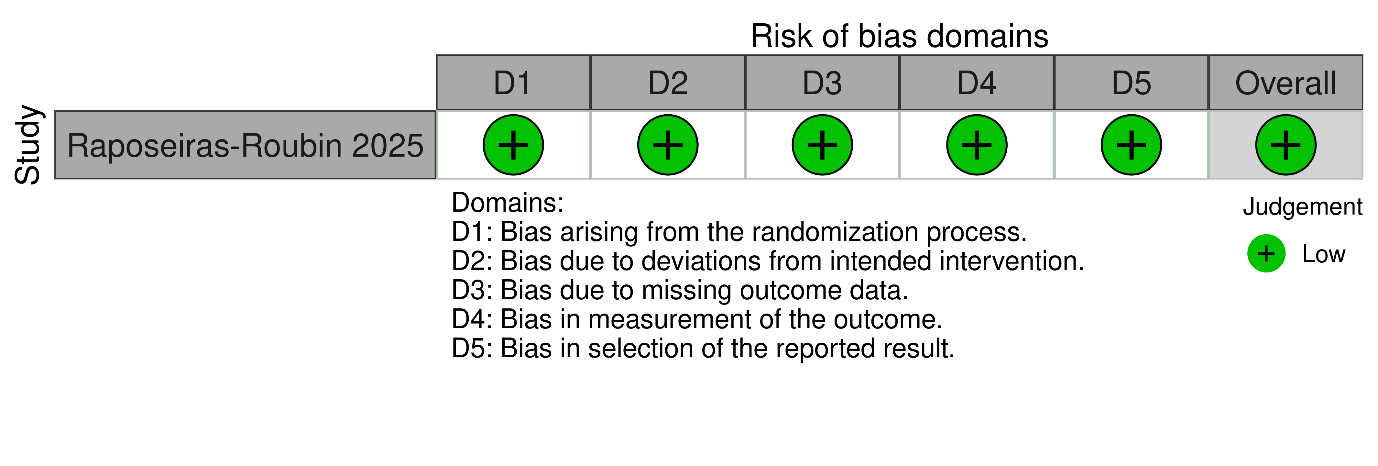


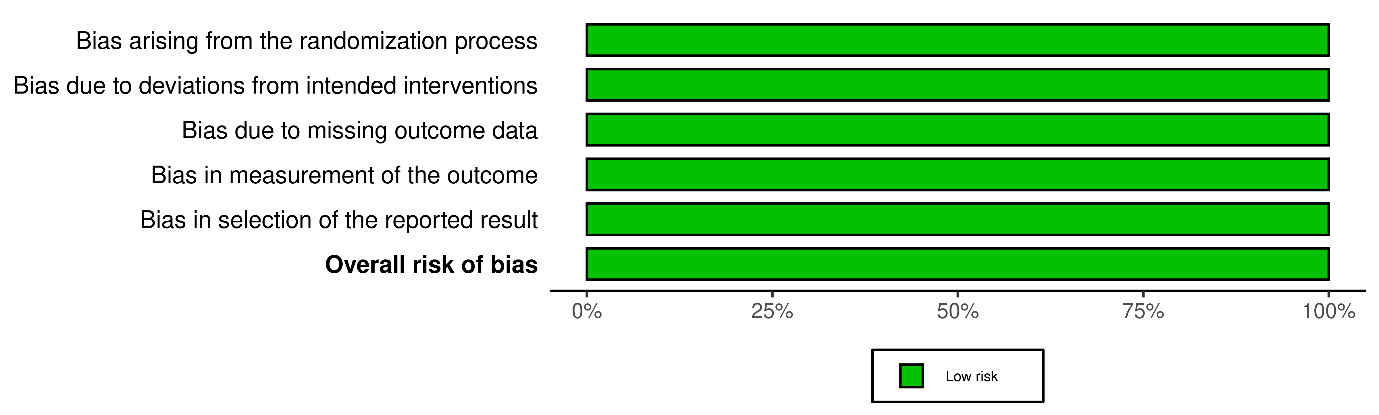


Displays the results of the risk of bias evaluation for each included study using a standardized assessment tool, highlighting potential methodological limitations.

**Supplemental Figure S2: Leave-One-Out Analysis Plots**

**MACE**


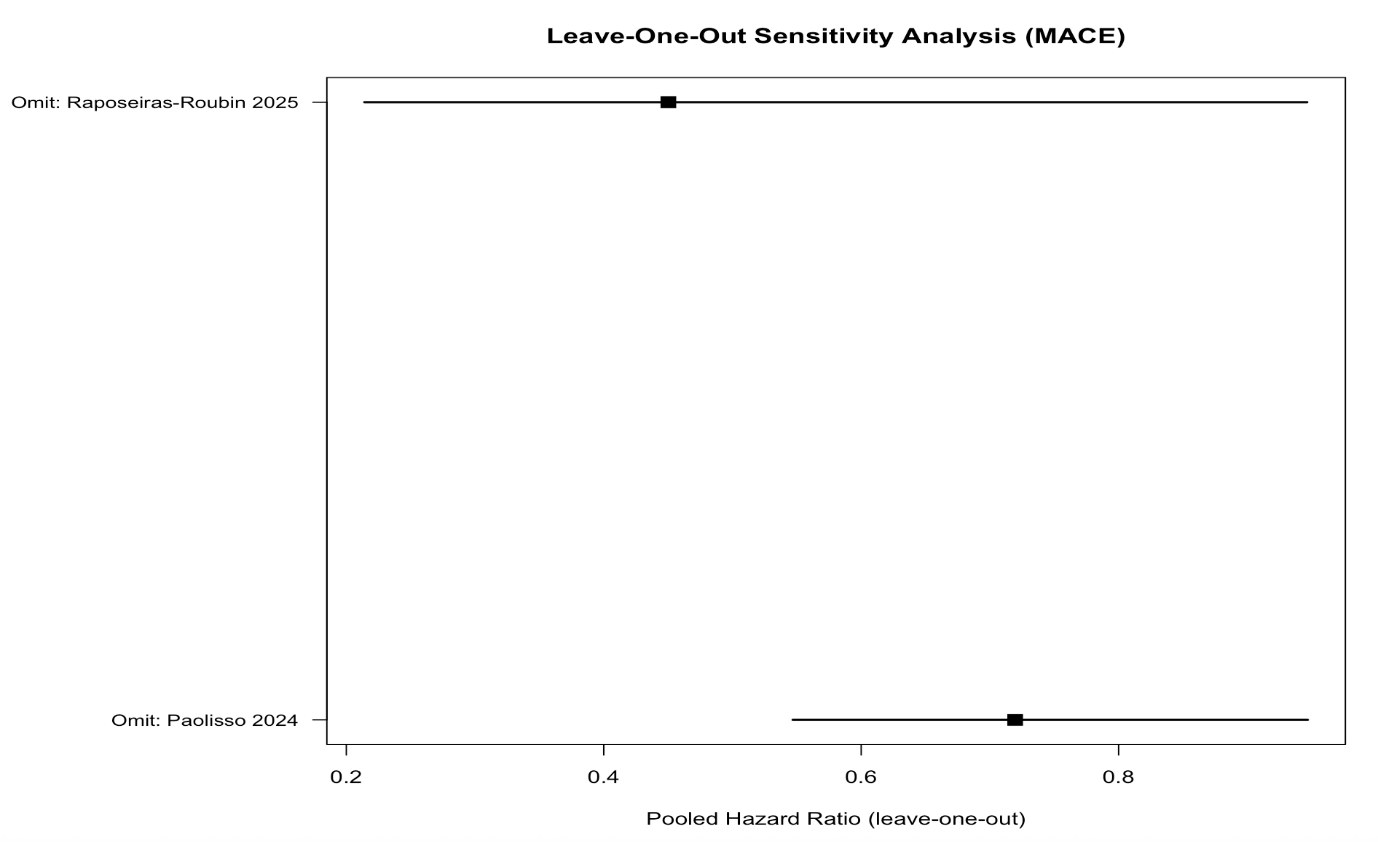


**Leave-one-out plot: Heart Failure Hospitalization**


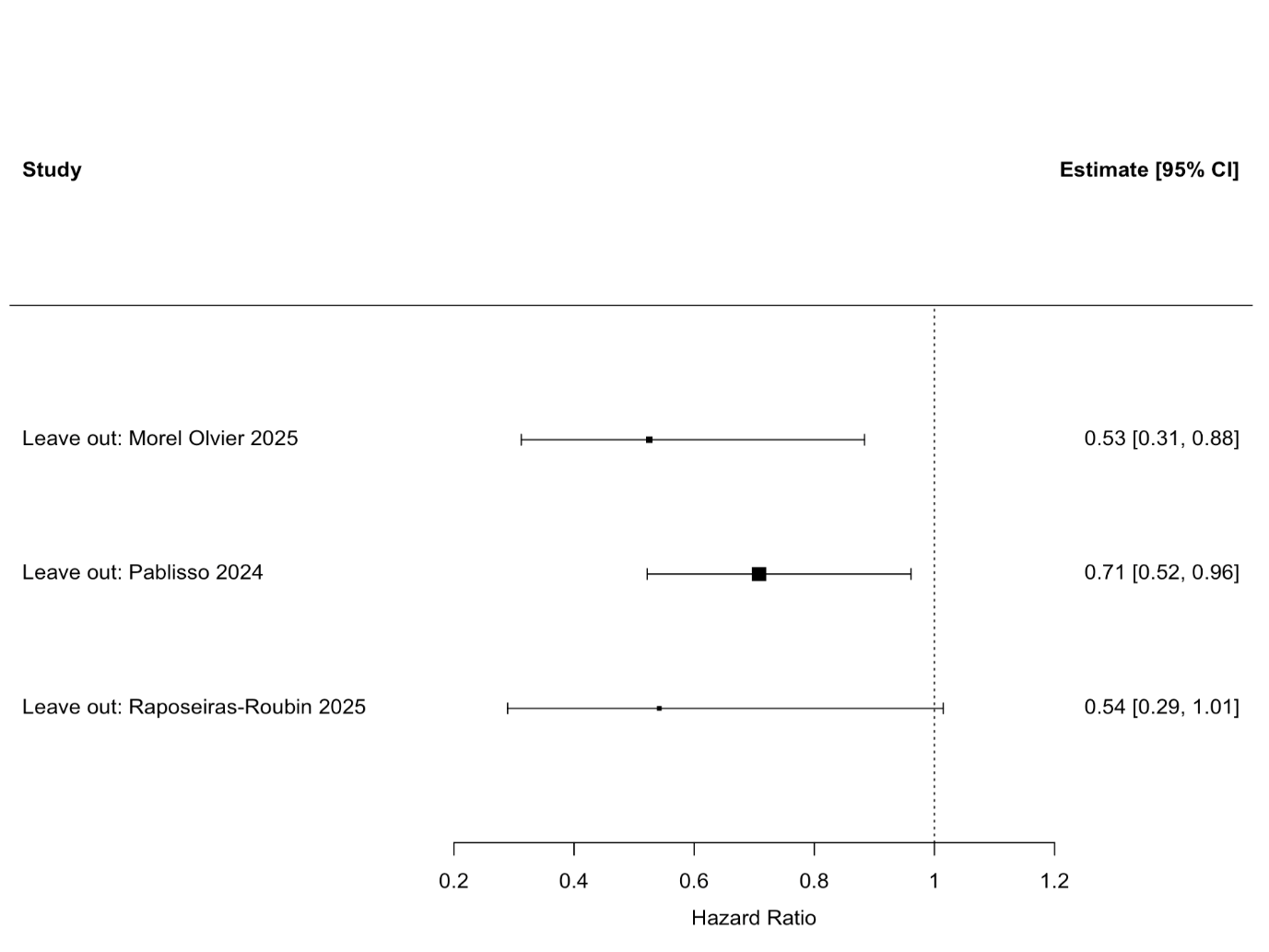


**Leave-one-out plot: Cardiovascular Death**


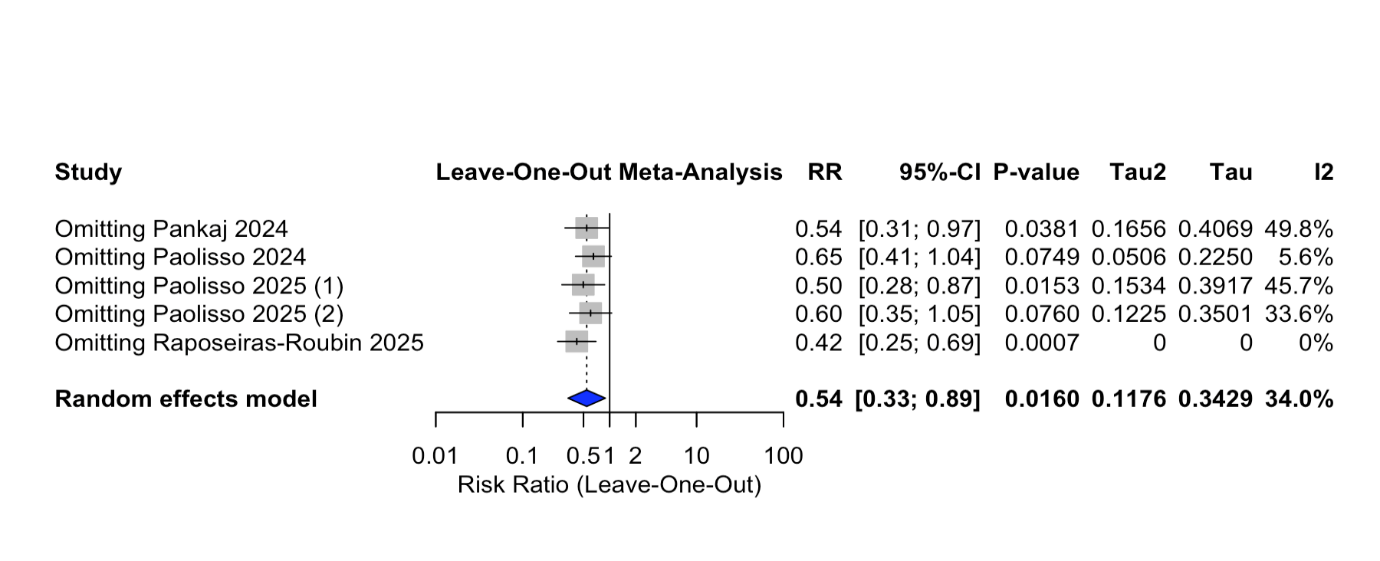


**Leave-one-out plot: All Cause Death**


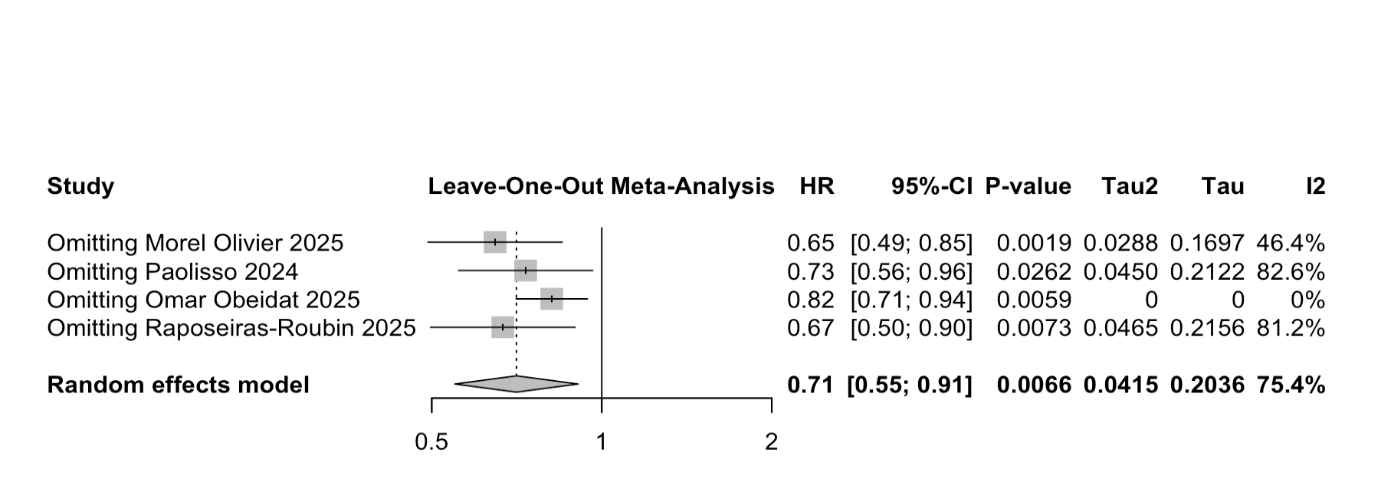


**Supplemental Figure S3: Sensitivity Analyses Plots for Heterogeneity Assessment (I^2^ =0)**

Heart Failure Hospitalization


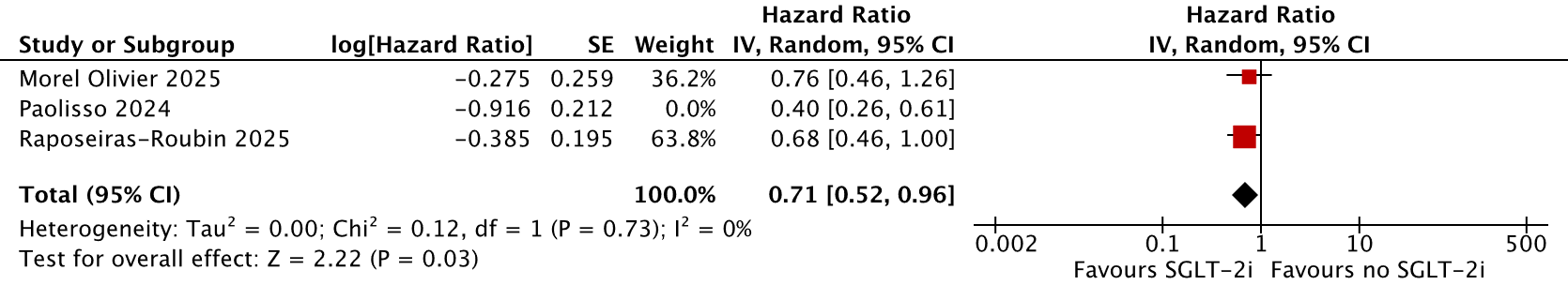


Cardiovascular Death


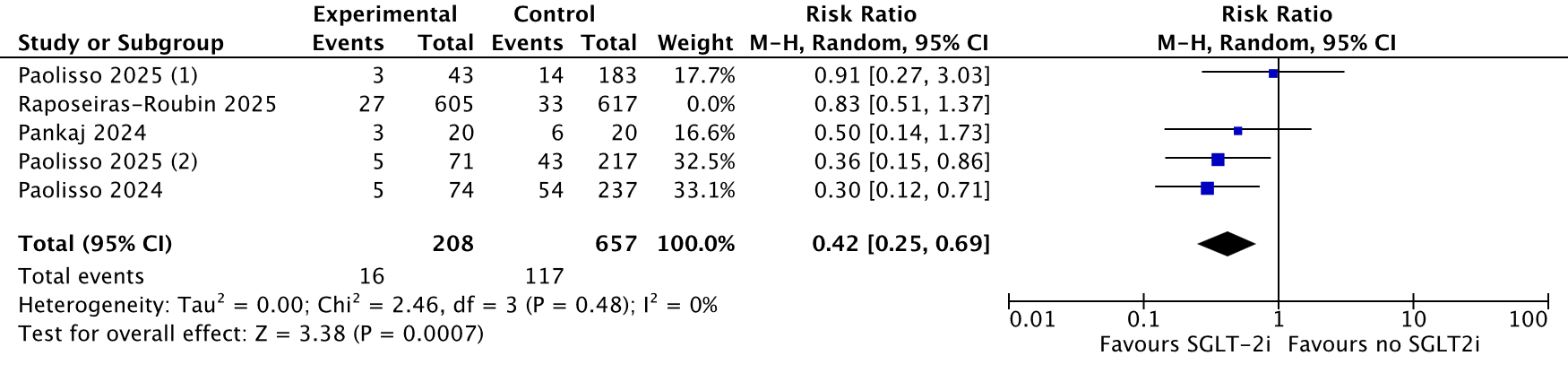


All Cause Death


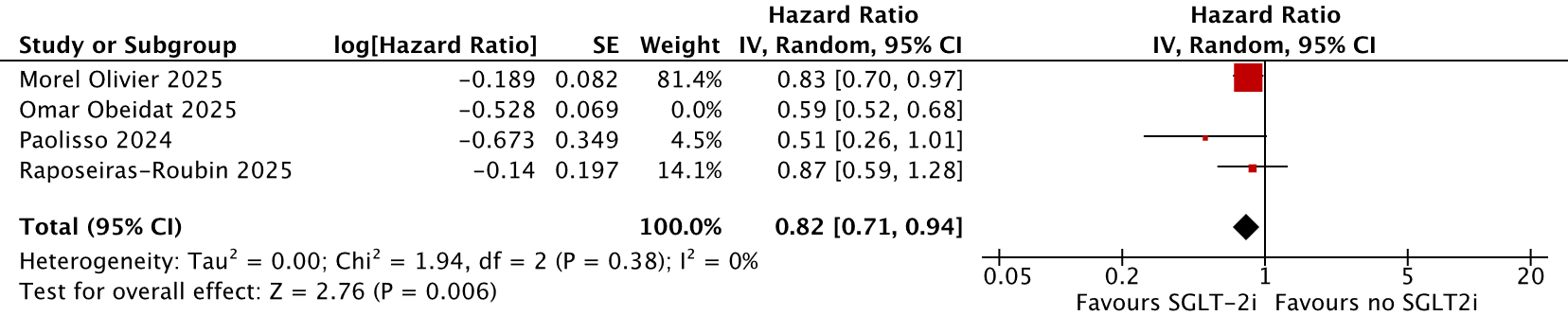


**Supplemental Figure S4: Funnel Plots for Publication Bias**

MACE


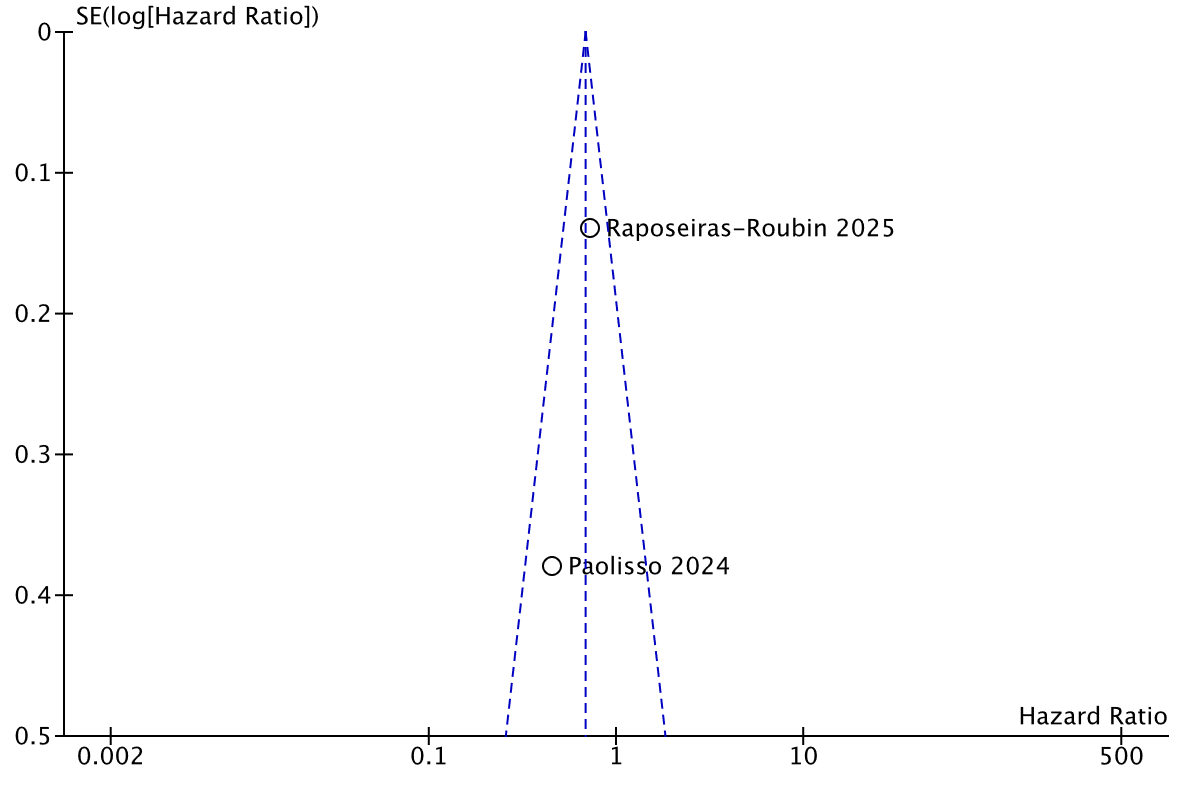


Heart Failure Hospitalization


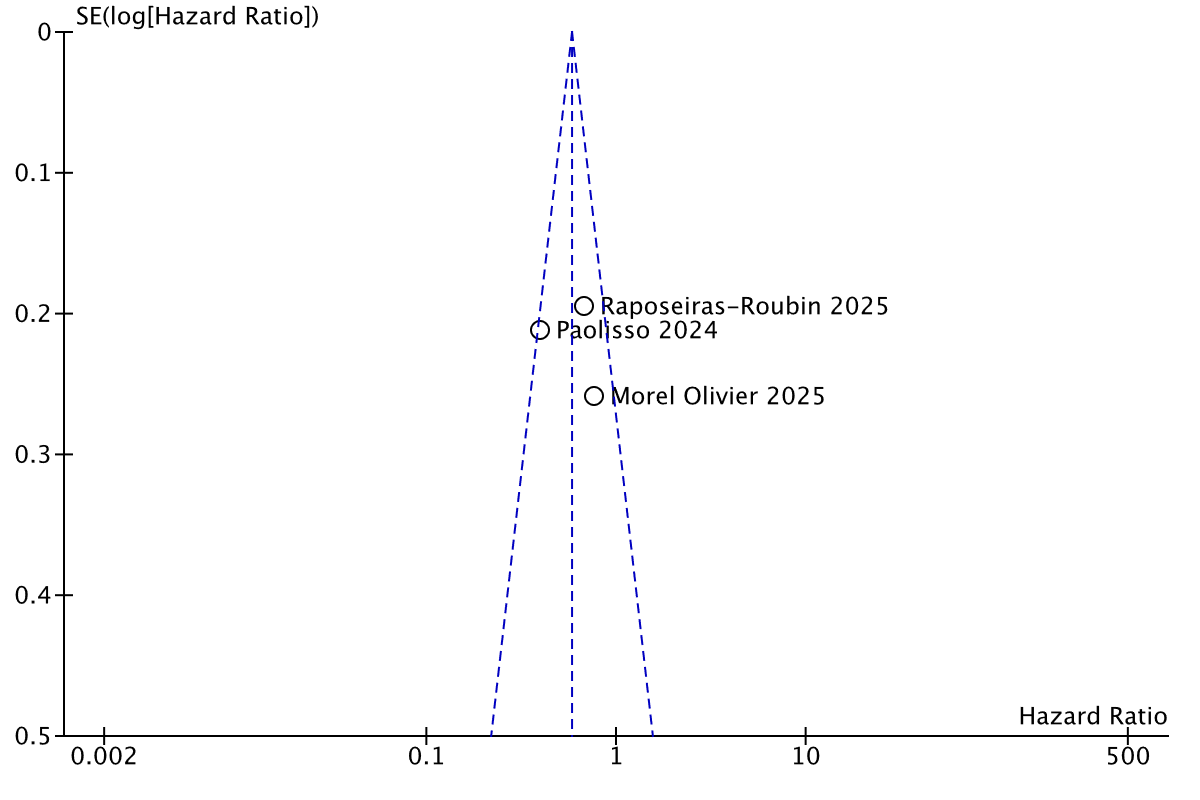


Cardiovascular Death


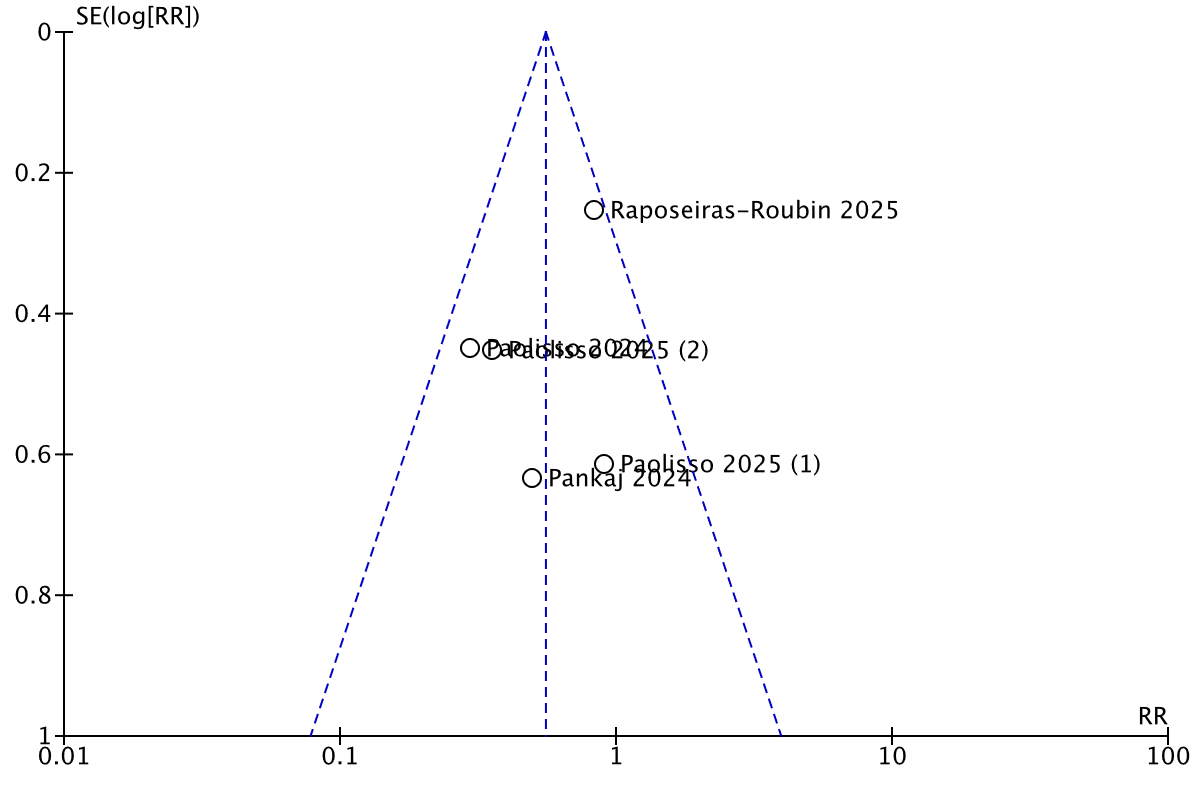


All Cause Death


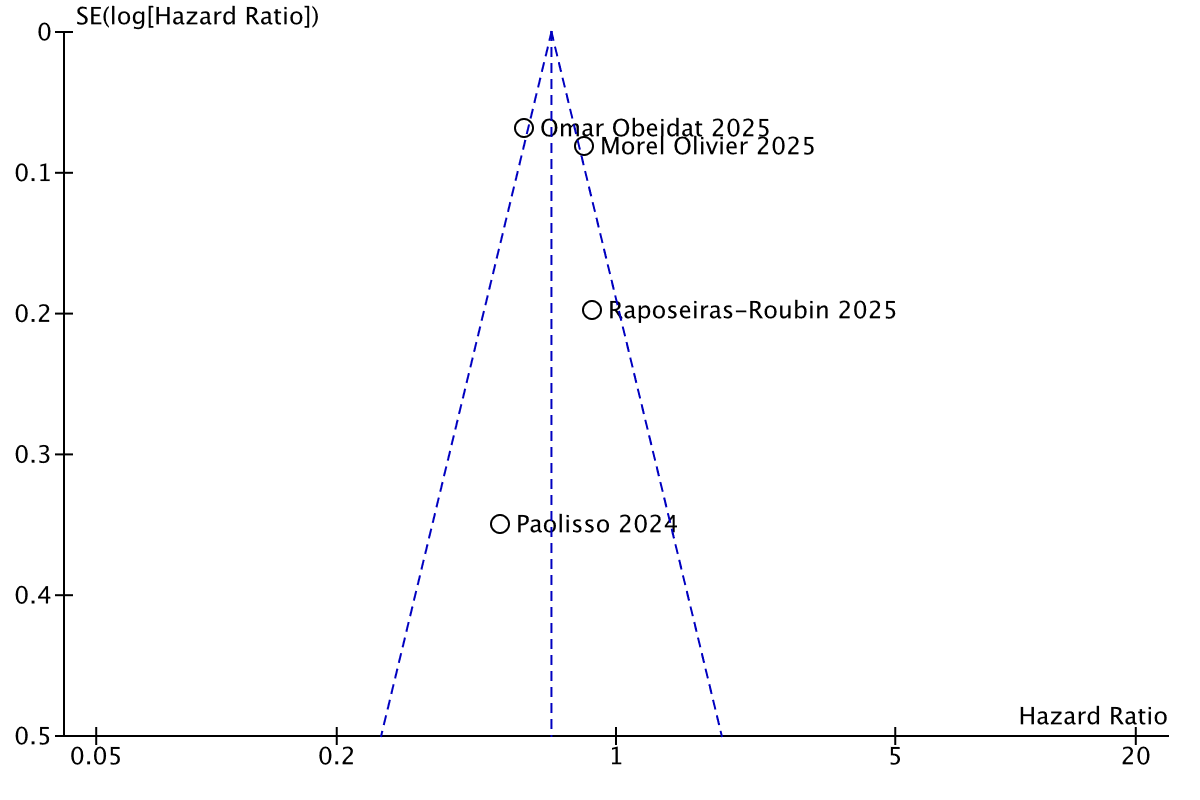


**Supplemental Figure S5: Sensitivity Analyses Plots After Excluding Pre-TAVI SGLT2i initiation study**

Cardiovascular death (exclusion of Pankaj 2024)


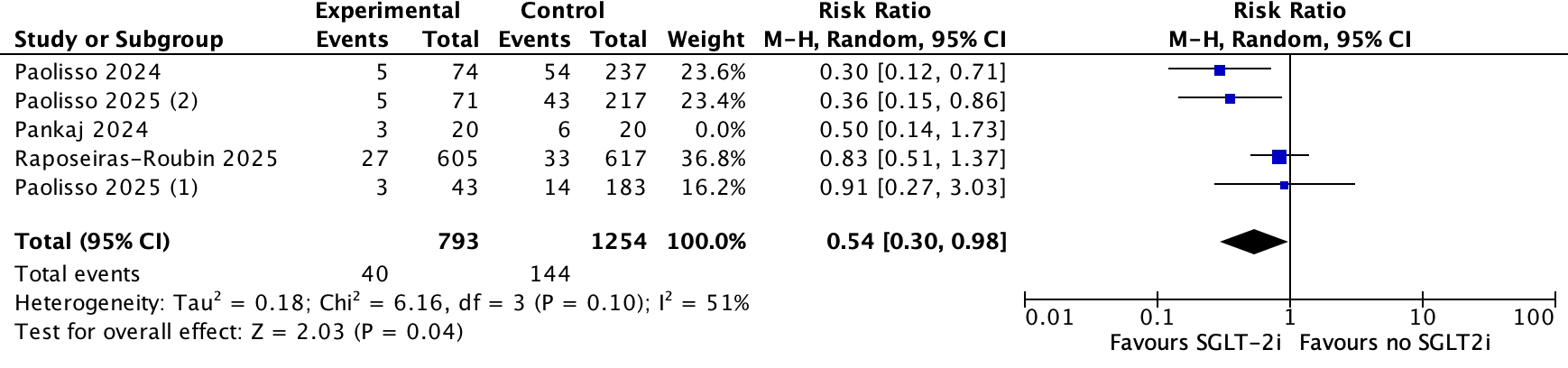


**Supplemental Figure S6: Sensitivity Analyses Plots After Hartung-Knapp Adjustment**


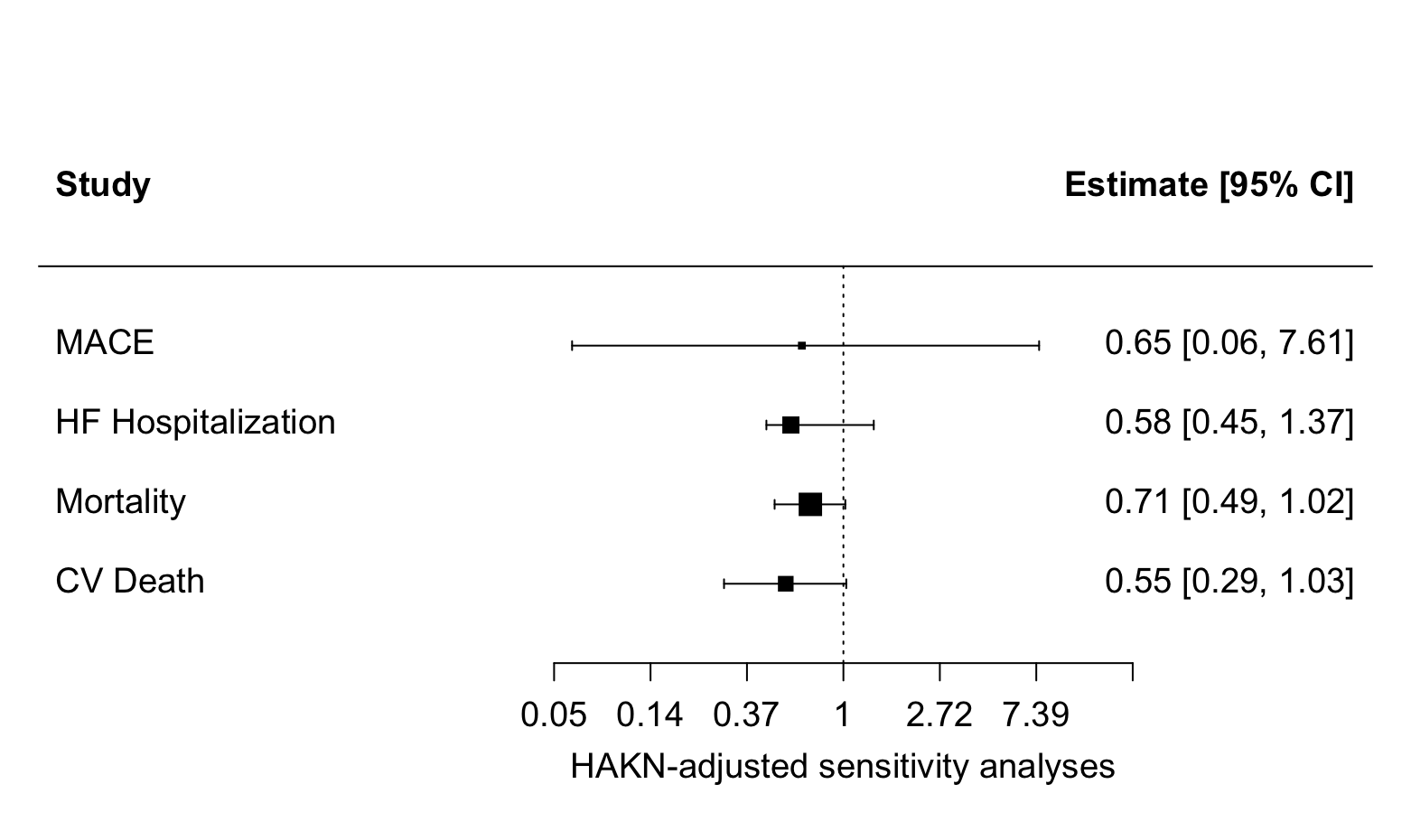

Supplement: Supplemental Tables 1 to 5 and Figures 1 to 6 [file mmc1.docx]
